# Are the numbers adding up? Exploiting discrepancies among complementary population models

**DOI:** 10.1002/ece3.1365

**Published:** 2014-12-24

**Authors:** Jennifer L Stenglein, Jun Zhu, Murray K Clayton, Timothy R Van Deelen

**Affiliations:** 1 Department of Forest and Wildlife Ecology, University of Wisconsin – Madison1630 Linden Drive, Madison, Wisconsin, 53706; 2 Department of Statistics, University of Wisconsin – Madison1300 University Avenue, Madison, Wisconsin, 53706; 3 Department of Entomology, University of Wisconsin – Madison1605 Linden Drive, Madison, Wisconsin, 53706; 4 Department of Plant Pathology, University of Wisconsin – Madison1605 Linden Drive, Madison, Wisconsin, 53706

**Keywords:** Bayesian inference, correction factor, gray wolves, hierarchical model, integrated population model, latent variable, population counts, radiotelemetry data, state-space model, survival analysis

## Abstract

Large carnivores are difficult to monitor because they tend to be sparsely distributed, sensitive to human activity, and associated with complex life histories. Consequently, understanding population trend and viability requires conservationists to cope with uncertainty and bias in population data. Joint analysis of combined data sets using multiple models (i.e., integrated population model) can improve inference about mechanisms (e.g., habitat heterogeneity and food distribution) affecting population dynamics. However, unobserved or unobservable processes can also introduce bias and can be difficult to quantify. We developed a Bayesian hierarchical modeling approach for inference on an integrated population model that reconciles annual population counts with recruitment and survival data (i.e., demographic processes). Our modeling framework is flexible and enables a realistic form of population dynamics by fitting separate density-dependent responses for each demographic process. Discrepancies estimated from shared parameters among different model components represent unobserved additions (i.e., recruitment or immigration) or removals (i.e., death or emigration) when annual population counts are reliable. In a case study of gray wolves in Wisconsin (1980–2011), concordant with policy changes, we estimated that a discrepancy of 0% (1980–1995), −2% (1996–2002), and 4% (2003–2011) in the annual mortality rate was needed to explain annual growth rate. Additional mortality in 2003–2011 may reflect density-dependent mechanisms, changes in illegal killing with shifts in wolf management, and nonindependent censoring in survival data. Integrated population models provide insights into unobserved or unobservable processes by quantifying discrepancies among data sets. Our modeling approach is generalizable to many population analysis needs and allows for identifying dynamic differences due to external drivers, such as management or policy changes.

## Introduction

Monitoring wildlife populations enables the estimation of key demographic parameters that inform conservation decisions. Patterns and drivers of population dynamics can be inferred by complementary models reflecting different paradigms and different sources of data (Sibly and Hone 2002). However, complementary models of the same phenomenon may not agree, resulting in a discrepancy. In terms of rigor, combining density-dependent, demographic, and mechanistic models and exploring emergent discrepancies is more holistic, which can improve prediction, and provides better understanding of potentially complex responses to ecological context, policy or human interventions.

Assuming that annual population counts are reliable, discrepancies from an unobserved process could result from multiple factors. For example, a discrepancy at low population sizes in a logistic growth model could result from underestimated emigration or overestimated survival rates, an Allee effect, or occurrence of unaccounted for exploitation (known as cryptic poaching; McCarthy 1997; Hoyle and Maunder 2004; Hurford et al. 2006; Liberg et al. 2012). In addition, survival data based on fates of individuals can carry unobserved effects into a model when individuals are lost to follow-up, resulting in unknown fates (Stenglein 2014). Commonly, these individuals are censored from the analysis at the date they were last observed (Klein and Moeschberger 2003). Traditional methods of survival analysis assume that censoring events are statistically independent of mortality events, but this assumption is often violated in wildlife studies because a loss to follow-up may be associated with an increased likelihood of death (Murray 2006). Failure to accommodate violation of this assumption usually leads to positive biases in survival estimates (Klein and Moeschberger 2003; Stenglein 2014). Whatever the circumstances behind a discrepancy, the unobserved process may be modeled to correct a population dynamics model.

Integrated population models (IPMs) provide a promising approach for detecting discrepancies in time series of population counts, improving estimates of demographic rates, and quantitating uncertainty in combined models of population counts and demographic rates (Besbeas et al. 2002; Brooks et al. 2004; Tavecchia et al. 2009). IPMs combine multiple submodels with shared parameters into a common model and are most useful in long-term observational studies where supplementary data have been collected simultaneously (Schaub and Abadi 2011). Population counts and annual recruitment and survival rates are the most basic components of a population dynamics study. Because population counts are an accounting of the annual recruitment and survival of the population, these data sets provide some redundant information which is helpful for correcting possible biases in one or the other metric. However, data analysts often estimate recruitment and survival rates independently without including population count information (e.g., Coulson et al. 2001). Here, in an IPM, we use this redundancy to better estimate all demographic processes, including those that are unobserved (Brooks et al. 2004; Abadi et al. 2010b; Schaub and Abadi 2011).

Using the IPM framework (Brooks et al. 2004; Tavecchia et al. 2009; Abadi et al. 2010b), we develop a Bayesian hierarchical model for integrating multiple sources of data and shared parameters and therefore provide a flexible and informative approach to quantify unobserved processes such as cryptic poaching rates, informative censoring, observation bias, complex density dependence, or human actions on the population as a result of management changes. We demonstrate this flexible, general method using commonly collected population data and provide an example of an IPM for estimating correction in the population growth of the gray wolf (*Canis lupus*) population in Wisconsin, USA (1979–2012).

## Materials and Methods

### Model development

**Figure 1:**
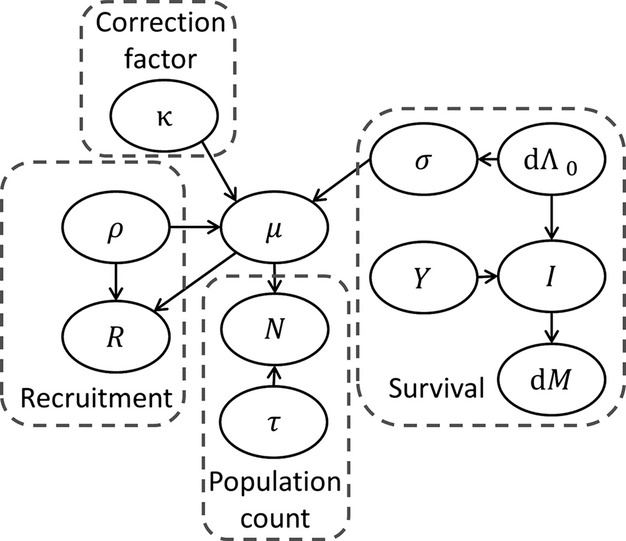
Directed acyclic graph (DAG) of the observation and process components of an integrated population model for the population dynamics of wolves in Wisconsin, USA. The notation matches the notation found in the text.

The data comprise time series of annual population counts, annual recruitment data, and telemetry-based survival data, which are common in wildlife monitoring and research. We develop a Bayesian hierarchical IPM that has three components: models for the observed data, models for the underlying population process, and prior distributions of the model parameters (Fig.1).

#### Observation models

The population count, *N*_*t*_, in a given year *t* is modeled by a log-normal distribution where *t *=* *1, 2, …, *T* for a total of *T* number of years. That is, *N*_*t*_ ∼ lognormal (*μ*_*t*_, *v*^2^), where *μ*_*t*_ is the true population size in year *t*, and *v* is the standard deviation of measurement error that may either be known or can be estimated, both on the log scale (Liberg et al. 2012).

The annual count of new recruits *R*_*t*_ born into the population in year *t* is modeled using a binomial distribution *R*_*t*_ ∼ binomial(*μ*_*t*−1_, *ρ*_*t*_) with recruitment rate, *ρ*_*t*_, from the population size in the previous year, *μ*_*t*−1_, where *ρ*_*t*_ is the true proportion of new recruits that are born into the population and survived until the annual count of *R*_*t*_ for *t *=* *2, 3, …, *T*. In the normal approximation to the binomial, we model 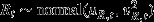, where *μ*_*R*,*t*_ = *μ*_*t*−1_ × *ρ*_*t*_ and *ν*_*R*,*t*_ = (*μ*_*t*−1_ × *ρ*_*t*_ × (1 − *ρ*_*t*_))^1/2^ are the mean and standard deviation of the normal distribution.

The annual survival records *i* where *i* = 1, 2, …, *n* and *n* is the number of records are modeled with a Cox regression (Kalbfleisch 1978; Clayton 1991). In counting process notation, *M*_*i*_(*s*) is the count of the number of events that occur up to time *s*, d*M*_*i*_(*s*) is the increment of the counting process over some small time interval, [*s*, *s *+ d*s*). The observed data are in triplet *X* = {*a*, *b*, *δ*}, where *a* is the start of the record, *b* is the end of the record, and *δ* is the indicator as to whether the end of the record was a death event (*δ* = 1) or a censoring event (*δ* = 0). The observed process *Y*_*i*_(*s*) takes a value of 1 if the triplet *X* is included in the data up to time *s* if *a* < *s* and *b* ≥ *s*, and *Y*_*i*_(*s*) = 0 otherwise. The increment of the counting process d*M*_*i*_(*s*) for some time *s* jumps when the record *i* is included in the data (*Y*_*i*_(*s*) = 1) and experiences the event (*δ* = 1). We model d*M*_*i*_(*s*) as independent Poisson random variables with means equal to the intensity *I*_*i*_(*s*) over the time interval [*s*,*s *+ d*s*): d*M*_*i*_(*s*) ∼ Poisson (*I*_*i*_(*s*)d*s*) (Clayton 1991).

#### Process models

In the lognormal model for the population counts, we assume that the mean process, *μ*_*t*_, is equal to the log of the population size *μ*_*t*−*1*_ in the previous year *t*−1 multiplied by the sum of a recruitment rate (*ρ*_*t*_), a survival rate (*σ*_*t*_), and a correction factor (*κ*_*t*_):

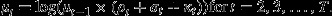


We assume a closed population where the immigration rate and emigration rate sum to zero and are not included in the model (Gotelli 1995). The correction factor *κ*_*t*_ is a rate that estimates the annual discrepancy among the annual population counts and survival and recruitment rates (Fig.1).

We compared four different models for recruitment rate. The annual recruitment rate, *ρ*_*t*_, is the success probability of a binomial distribution for the annual count of new recruits through birth, *R*_*t*_, and we let *ρ*_*t*_ ∼ beta (*α*_1_, *α*_2_) with parameters *α*_1_ and *α*_2_. Alternatively, we give a functional form for the relationship between *ρ*_*t*_ and log (*μ*_*t*−1_). A linear relationship is *ρ*_*t*_ = *β*_0_ + *β*_1_ × log (*μ*_*t*−1_) and a quadratic relationship is *ρ*_*t*_ = *β*_0_ + *β*_1_ × log (*μ*_*t*−1_) + *β*_2_ × (log (*μ*_*t*−1_))^2^. These alternative relationships of *ρ*_*t*_ related to population size allow for some simple tests of density dependence (Gotelli 1995).

In the model for the survival records, we model the intensity increment over a small time period, *I*_*i*_(*s*)d*s*, as the product of the integrated baseline hazard function, *dΛ*_*o*_(*s*), and whether record *i* is in the risk set, *Y*_*i*_(*s*), during that time period, [*s*,*s *+ d*s*): *I*_*i*_(*s*)d*s* = *Y*_*i*_(*s*) × dΛ_0_(*s*). We assume constant integrated baseline hazards within each [*s*,*s *+ d*s*) to have a proportional hazards form (Ibrahim et al. 2005). To compare alternative models for density dependence in the survival process, we give *I*_*i*_(*s*)d*s* some functional form for the relationship between the intensity increment and the standardized log (*μ*_*t*−1_). A log-linear relationship is 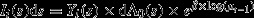, and a log-quadratic relationship is 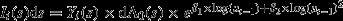. The annual survival rate, *σ*_*t*_, is the exponential of the negative sum of the increment in the hazard function across *t* raised to the power of the functional form relationship. For example, the linear functional form for survival is 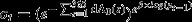 (Klein and Moeschberger 2003; Spiegelhalter et al. 2003).

#### Prior distributions

For the priors in the population model, we use a wide, uniform prior on the standard deviation in the lognormal model for the population count: *v* ∼ uniform (0, 100) (Gelman et al. 2003). We also use a vague, uniform prior on the correction factor: *κ*_*t*_ ∼ uniform (−1, 1) so that we do not restrict the correction factor *κ*_*t*_ to be positive or negative in any year *t*.

In the beta-distribution for the recruitment rate *ρ*_*t*_, we take the parameters *α*_1_ = 1 and *α*_2_ = 1 as on the hyperpriors for the rate *ρ*_*t*_. When including the linear model, we model 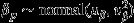 for *p* = 1, 2, …, *P* where *P* is the number of parameters, and the hyperparameter *μ*_*β*_ is the mean and equal to 0, and *ν*_*β*_ is the standard deviation and equal to 100 (Gelman et al. 2003).

We model the incremental integrated baseline hazard function with independent Gamma priors: 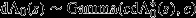, where 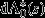 is a reasonable guess at the mean of the process: 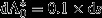 where d*s* is the size of the time interval. The parameter *c* = 0.001 is a positive real number that reflects the level of confidence in 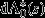 with a smaller value corresponding to lower confidence (Kalbfleisch 1978).

To evaluate the methodology in the model development section for detecting a correction factor, we conducted a simulation study (SeeAppendix S1 in Supporting Information).

### Case study: Wolf population growth in Wisconsin

#### Wisconsin wolf population

**Figure 2:**
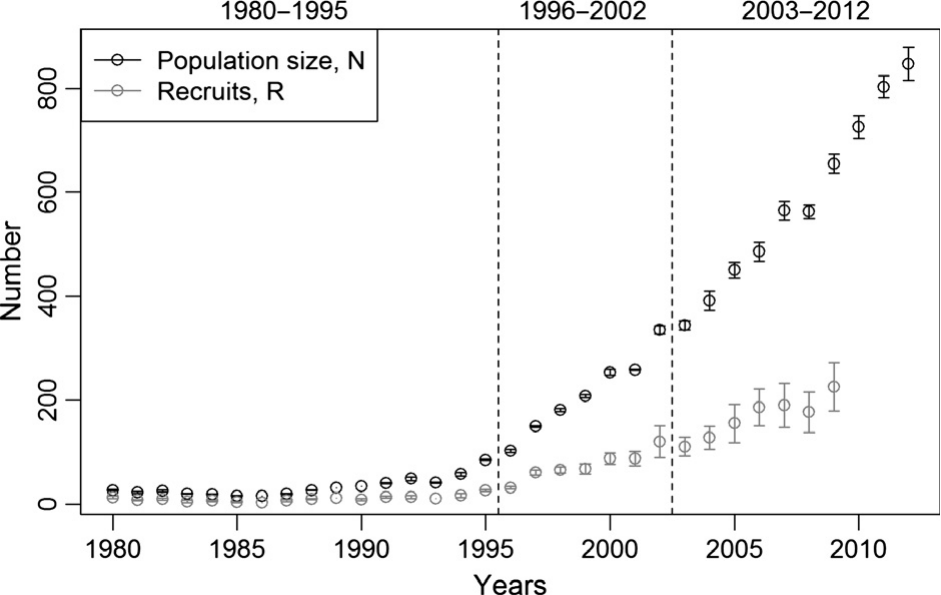
Wisconsin, USA estimated wolf population mean size (and range) and estimated mean number of new recruits (and range) from 1980 to 2012 with three recovery periods denoted by vertical dotted lines.

The Wisconsin wolf population increased from about 20 individuals in the early 1980s to >880 individuals in 2012 (Fig.2). The population grew slowly from 1980 to 1995 at which point the winter count surpassed the endangered status of 80 wolves (Wydeven et al. 2009). Since 1995, the wolf population increased dramatically, and management policy changed with respect to the degree to which managers may kill wolves to address depredation problems. Hence, policy changes and population growth interacted to define three recovery periods (Fig.2). During 1996–2002, wolves were listed as endangered under the US Endangered Species Act and protected from all hunting and trapping. In 2003, wolves were downgraded to threatened status and lethal control actions by agency specialists were allowed on wolves that were seen as livestock and human safety risks (Ruid et al. 2009; Wydeven et al. 2009). The period 2003–2012 was dominated by this on-again and off-again lethal control management in response to lawsuits seeking to vacate delisting decisions (Olson 2013).

Each winter, the Wisconsin Department of Natural Resources (WDNR) counted the number of wolves, estimated the number of new recruits, and radiocollared and tracked individual wolves. The WDNR made the annual population counts through snow tracking by volunteers and agency personnel, direct observation, photographs from wildlife cameras, and by aerial counts of packs containing radiocollared individuals (Wydeven et al. 2009). Annual winter counts were reported as ranges (lower bound is the minimum count) in the WDNR annual reports, and we used the midpoint in each year as our count. We calculated measurement error as ¼ the difference between the high and low counts in each year and took the average among the years as *v*.

The WDNR reported the number of new recruits each winter in 1980–2009 as a range, and we used the midpoint of the range as the recruitment data in our model (Fig.2). We assigned survival records with a start time, end time, and censoring indicator to the 422 radiocollared wolves that were captured during 1979–2011 and survived at least until 1 September of their birth year (4–5 months old; see Wydeven et al. 2009 for details on capturing and radiocollaring methods). Including wolves that are >4 months old help to meet the survival analysis assumption that survival times are independent because Wydeven et al. (2009) found no difference in survival for pups (collared at 3–6 months old), yearlings, and adult wolves in Wisconsin from 1979 to 2003. Independence among data set is required for the construction of the joint likelihood in an IPM, and our data sets are not completely independent (Besbeas et al. 2002). Population counts and the number of new recruits are partially determined from aerial surveys of packs containing radiocollared individuals. However, the dependency among these data sets is minimal, and parameter estimates from IPMs are fairly robust to violation of the assumption of independence (Abadi et al. 2010a).

We assumed a separate correction factor, *κ*, for each of the three recovery periods, and let *κ* be constant within each recovery period (Fig.2).

#### Density dependence in recruitment and survival

Prior to running the integrated population model, we selected a set of functional forms for the relationship between the annual recruitment rate *ρ*_*t*_ and standardized (subtracted the mean from each value and divided by the standard deviation) log (*N*_*t*−1_) and the annual survival rate *σ*_*t*_ and standardized log (*N*_*t*−1_) from the following: (1) no relationship (i.e., constant survival or recruitment rates), (2) a linear relationship, (3) a quadratic relationship, and (4) two lines that changed at year *t*, for *t *=* *3, 4, …, 28, 29 (i.e., a change-point model, Chappell 1989). We expected some form of density dependence in the recruitment and survival processes because of the reduced population growth in the early years of the time series and a leveling off of growth in the most recent years (Van Deelen 2009). However, survival rates have been relatively constant during wolf recovery (Wydeven et al. 2009). Therefore, we did not expect to detect a density-dependent effect in the survival process, and we did expect to detect a density-dependent effect in the recruitment process with lower recruitment at the beginning and end of the time series, and a maximum recruitment associated with an intermediate density. We determined the best functional form for the recruitment process and the survival process based on which had the lowest Bayesian information criterion (BIC), and we assumed that models within 2 BIC units of each other were equivalent. We ran 3 Monte Carlo Markov Chains (MCMC) for 5000 burn-in iterations followed by 5000 iterations to estimate deviance for BIC calculation. We tested for proportionality in the predictors because of the proportional hazards assumption with a graphical check and statistical test of whether a time-dependent covariate interacting with the predictor was different from 0 for the best survival model if the best survival model included covariates (Klein and Moeschberger 2003).

#### Model specifications

After identifying the functional form for recruitment and survival based on BIC, we fit a model with three correction factors that were constant within each recovery period (1980–1995, 1996–2002, and 2003–2011). In the population count model and the recruitment model, we used *N*_*t*−1_ instead of *μ*_*t*−1_ in the time series to improve the chain convergence.

We performed model assessment including external validation, posterior predictive checking, and sensitivity analysis (Appendix S2). For external validation, the observed population size in 2012 was left out and we predicted it from the mean of the posterior predictive distribution. We judged our model to provide reasonable inference if the mean of the posterior prediction was within the observed wolf population size range in 2012. We performed graphical posterior predictive checks of estimated population counts from the model compared to what was observed, and we graphed the residuals to look for patterns that would indicate poor model fit. Finally, we performed a sensitivity analysis on our choice of priors (Appendix S2).

We ran three MCMC chains for 10,000 iterations after discarding the first 10,000 iterations as burn-in using program JAGS (Plummer 2003) accessed through program R (R developement Core Team 2013), package “rjags” (Plummer 2011). We assessed convergence using visual inspection of mixing in the chains, univariate potential scale reduction factors (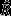, hereafter; Gelman and Rubin 1992), and the multiple potential scale reduction factor (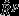, where *p* is the number of parameters; Brooks and Gelman 1998). By convention, convergence is generally judged to be attained when upper 97.5% confidence limits of the 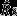 and 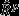 are close to 1, and here, convergence was declared if the upper 97.5% confidence limits of all 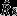 and 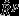 were <1.1.

## Results

Individual wolves associated with 1–10 yearly survival records depending on the number of years that they were in the study. Median number of survival records was 2. The functional form for the survival process with the lowest BIC was the constant model indicating no density dependence. This baseline hazard model was >3 BIC points lower than the next best functional form model. The functional form for the recruitment process with the lowest BIC were the change-point models that changed after years 14, 15, 17, or 18, and all of these models were >2.2 BIC points lower than the next best model. We used the model with a change-point after year 18 (very similar correction factor estimates for all best change-point models), and the formula for this model was as follows:

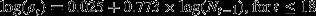


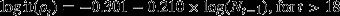


The evidence for a positive slope of the line for *t* ≤ 18 was 100% (proportion of posterior that was >0), indicating an increasing relationship of recruitment rate and population size when the wolf population was ≤150 wolves. The evidence for a negative slope of the line for *t* > 18 was 69.0% (proportion of posterior that was <0).

The MCMC algorithm converged adequately. The upper 97.5% estimates of 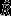 were 1 for all parameters, and the overall 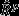 = 1. The mean of the posterior predictive distribution to estimate the population count in 2012 was 878, which was within the observed population size in 2012 of 815–880 wolves. Therefore, we concluded that the inferences from our model made sense (Appendix S2). Also, 48.4% of the time, the estimated population sizes in Wisconsin from 1981 to 2011 were within the 95% posterior intervals of *μ*_*t*_ for *t* = 2, 3, …, 32, respectively. When the 95% posterior intervals for the estimated population sizes did not overlap the estimated population sizes in Wisconsin, they were not systematically overestimated or underestimated.

**Figure 3:**
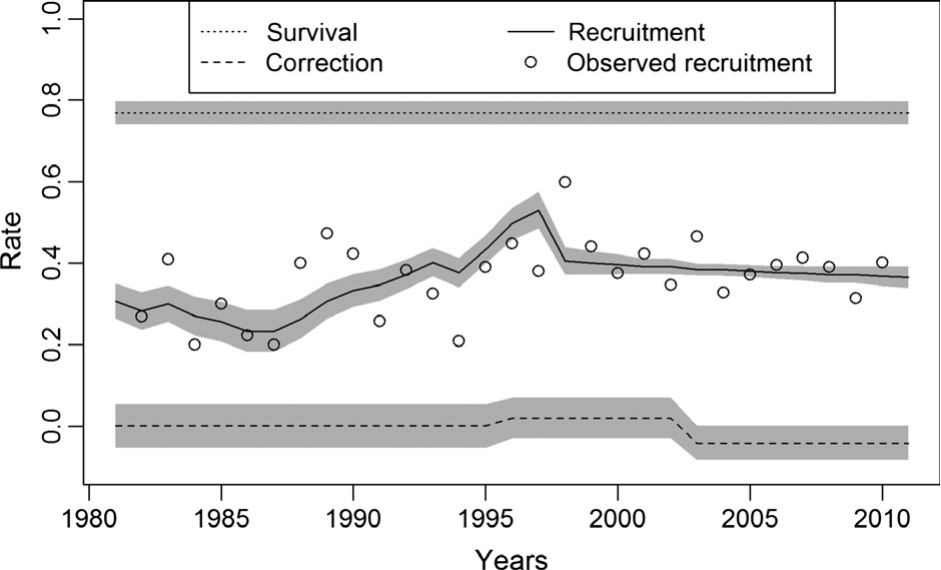
Observed recruitment and posterior estimates and 95% credibility intervals for annual recruitment, survival, and an estimated correction factor in the Wisconsin, USA wolf population from 1980 to 2012.

**Figure 4:**
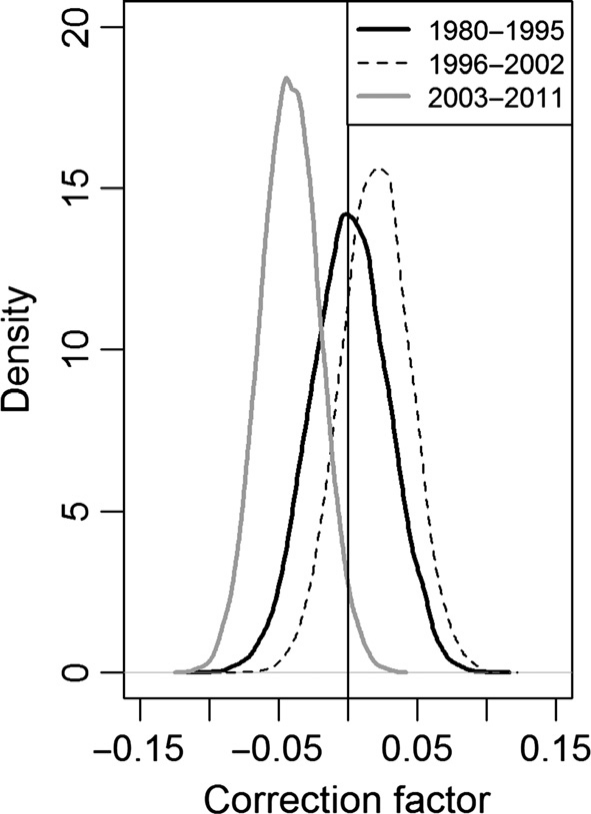
Posterior densities of the correction factors (*κ*) in the annual counts for three recovery periods in the Wisconsin, USA wolf population. The amount of the distribution left of the vertical line at 0 shows strength of evidence for a negative correction factor.

Estimated annual mean recruitment rate varied from 0.233 to 0.530, with the lowest recruitment estimated during early recovery (Fig.3). Estimated recruitment rate reached its maximum of 0.530 (SD = 0.023) in 1997 (Fig.3). On average, estimated recruitment rate was 0.013 lower in 1980–1995 compared to the average observed recruitment rates in that time period (Fig.3). The other time periods had estimated and observed recruitment rates that were within 0.005 of each other, on average. The average annual survival rate was estimated at 0.769 (SD = 0.014; Fig.3). The correction factors were estimated at 0.0002 (SD = 0.027) in 1980–1995, 0.020 (SD = 0.025) in 1996–2002, and −0.042 (SD = 0.022) in 2003–2011 (Fig.4). The evidence for an estimated net additional loss needed to explain the population dynamics was 50.1% in 1980–1995, 21.7% in 1996–2002, and 97.4% in 2003–2011 (proportion of posterior <0; Fig.4).

## Discussion

Unifying complementary models of population dynamics can improve our understanding of both observed and unobserved processes affecting population trend. For instance, when population counts and demographic projections differ, we can sometimes detect an unobserved process that reveals or suggests a more complete mechanistic understanding of the population's dynamics. To this end, we developed a general IPM to quantify a demographic discrepancy between population count data when supplemental survival and recruitment data were available. Through simulations, we showed that our model detected and recovered discrepancies that were distinct from sources of variation in recruitment, survival, and measurement error in the population counts, and therefore, the estimated discrepancies represented an unobserved process (see Appendix S1).

There are always opportunities to add complexity to a model, but we show how a simple IPM could unite data sets that are widely available for managed populations. Our model is accessible because we present it in a generalized form and give basic ideas for incorporating density dependence in demographic processes. The counting process notation appears complicated, but it is actually a straightforward technique to incorporate the radiotelemetry-based survival analysis into a Bayesian IPM (see Appendix S3). An extension for incorporating density dependence in the demographic processes would be to use a Gaussian process (Paciorek 2007) or spline function, such as low-rank thin-plate splines that have good mixing properties in a Bayesian analysis (Crainiceanu et al. 2005; Stenglein 2014).

We modeled Wisconsin wolf population dynamics with different patterns of density dependence in demographic components and a correction for population growth that varied with recovery period as defined by population growth and wolf management policy. Mean recruitment was more variable and increasing in 1980–1997 compared to the time period since 1997. Low recruitment at low density is consistent with an Allee effect impacting mate-finding ability when potential mates are sparse (McCarthy 1997; Stenglein 2014). Reduced and less variable recruitment at higher population sizes is evidence of density dependence in recruitment. However, we did not detect a density-dependent effect on the survival process, consistent with another analysis of the same data between 1979 and 2003 (Wydeven et al. 2009). As wolves in Wisconsin further saturate their preferred habitat, we may expect a density-driven reduction in survival (Mladenoff et al. 2009; Van Deelen 2009), although detecting this effect will require more years of data at these higher population sizes (Van Deelen 2009). Estimating density-driven changes separately in recruitment and survival provides insights into specific mechanisms that affecting population growth (Clutton-Brock and Coulson 2002; Bonenfant et al. 2009).

We found that additional loss was needed to reconcile the demographic processes with the population counts when the wolf population was growing at about 10% per year and the population increased from 344 to 803 wolves in 2003 to 2011. We included density-dependent changes in the demographic components of our model; therefore, it is unlikely that the discrepancy is explained by additional density-dependent mechanisms. Nevertheless, the largest estimated correction occurred in the most recent years and may partially reflect decelerating growth, as predicted by Van Deelen (2009). Our model predictions for the population size in 2012 were within range, but were high compared to the observed estimated growth in 2012, which further supports a density-dependent reduction in population growth. Emigration is another mechanism to potentially explain the discrepancy. We assumed population closure which effectively made immigration and emigration equal. Wisconsin wolves and the larger Great Lakes wolf population have evidently saturated much of their preferred habitat (Mladenoff et al. 1995, 2009; Van Deelen 2009). Concurrent with rangewide occupancy of prime habitat, we might suspect an increase in the number of Wisconsin emigrants, except that the neighboring populations in Michigan and Minnesota are also saturated and likely producing immigrants into Wisconsin at the same rate. Density dependence or emigration may explain some of the discrepancy in 2003–2011, but there are also other potential reasons for this discrepancy.

Radiotelemetry-based survival analysis may be plagued with a level of informative censoring that is difficult to quantify, but can have serious consequences by biasing estimated survival rates high (Murray 2006). Informative censoring occurs when individuals are lost to follow-up, and their loss is associated with the event of interest, in this case death. Unobserved poaching has been linked to a discrepancy in growth of the Scandinavian wolf population (Liberg et al. 2012), and it is a likely source for informative censoring in survival data. Our case study shows an average correction of 4.2% additional loss each year since 2003. Approximately 60% of Wisconsin's radiocollared wolves in the survival data set are censored and more than half of those have disappeared from the analysis without any indication of collar failure or dispersal (Stenglein 2014). Our estimated correction factor may give us the best information to date on a level of informative censoring in our radiotelemetry survival data. An average of 4.2% additional loss to explain the annual growth rate translates to informative censoring (death) associated with about 25% of the wolves among that disappeared (Stenglein 2014).

The greatest discrepancy that we detected was during 2003–2011, coincident with declining public attitudes toward wolves (Browne-Nunez et al. 2012). Attitude surveys of Wisconsin farmers and hunters revealed a fear of wolves, lack of empowerment toward wolf issues, and an increase in willingness to illegally kill a wolf from 2001 to 2009 (Browne-Nunez et al. 2012; Olson 2013; Treves et al. 2013). Inconsistent wolf management during 2003–2011 may have led to an increase in illegal killing that contributed to additional unobserved annual population loss. This IPM provides an important baseline prior to the beginning of an additional source of mortality for wolves in the form of a recreational harvest that began in 2012 and will likely continue.

Our case study with wolves illustrates that detecting discrepancies among complementary models improves our understanding of the potentially complex responses populations have to policy, human interventions, or other external factors. Similarly, analysts may be faced with the problem of known or suspected population processes that, while important, are unobservable in given sampling design or logistical constraints. Our modeling framework makes estimating those processes more attainable.

## Data Accessibility

- R code and models for simulations: uploaded as online supporting information, Appendix S1
- Integrated population model for use with JAGS: uploaded as online supporting information, Appendix S3
- Wolf survival data: uploaded as online supporting information, Appendix S3
- Wolf population count and recruitment data, 1980–2007: available from Wydeven et al. 2009
- Wolf population count data, 2008–2012, and recruitment data, 2008– 2009: available from WDNR annual reports online at www.dnr.wi.gov

## Supporting Information

**Appendix S1** Detailed description of simulation study and assessment.

**Appendix S2** Model assessment.

**Appendix S3** Bayesian integrated population model with correction factor.
