# Supplementary material for: Are the numbers adding up? Exploiting discrepancies among complementary population models
Source: Ecol Evol. 2014 Dec 24;5(2):368–76. doi: 10.1002/ece3.1365 (PMC4314269; doi:10.1002/ece3.1365)
Supplement: Supplementary file 1 [file ece30005-0368-sd1.docx]

**Appendix S1.** Detailed description of approach, assessment, and R code for simulation study to detect a correction factor with an integrated population model (IPM)

**Approach**

To evaluate the methodology in the model development section for detecting a correction factor, we conducted a simulation study. In particular, we assessed the effects of magnitude of the true correction factor (*κ*), length of time series (*T*), and amount of missing data on the model performance. We varied levels of the correction factor (*κ* = 0, 5%, and 10% annual additional loss from the population), the number of years of data (*T* = 10, 20, or 30 years), and amount of missing data (all data, 20% missing recruitment and count data). In program R, we simulated a dataset for each of these 18 combinations 100 times and fitted the integrated population model (IPM). We constructed 31 year, complete datasets from the model:

$$N_{t}\sim normal\left( \mu_{t},2^{2} \right)$$

$$\mu_{t}=N_{t-1}\times(\sigma_{t}+\rho_{t}+\kappa)$$

where *t* was an index of years from *2,3,…,T-1,T*, and we set *T* at 31 years. We set *κ* = 0, -0.05, or -0.1. We used a simpler model in simulations than we described in the model development and case study portions of the paper because we did not need to estimate annual survival rates to assess our approach. Instead, we assumed both recruitment and survival were drawn from normal distributions reflecting reasonable mean and variance estimates ([Wydeven *et al.*, 2009](#_ENREF_2)). We let the mean of the recruitment rate change after year 6 and the mean of the survival rate change after year 18. Therefore,

$$\rho_{t}\sim normal\left( \mu_{\rho1},\tau_{\rho,t} \right), for t=1, 2, \ldots, 6$$

$$\rho_{t}\sim normal\left( \mu_{\rho2},\tau_{\rho,t} \right), for t=7, 8, \ldots, T-1, T$$

$$\sigma_{t}\sim normal\left( \mu_{\sigma1},\tau_{\sigma,t} \right), for t=1, 2, \ldots, 18$$

$$\sigma_{t}\sim normal\left( \mu_{\sigma2},\tau_{\sigma,t} \right), for t=19, 20, \ldots, T-1, T$$

where $\mu_{\rho1}=0.35, \mu_{\rho2}=0.45, \mu_{\sigma1}=0.8, \mu_{\sigma1}=0.7$, and $\tau_{\rho,t}= \tau_{\sigma,t}={0.05}^{2}$.

We used an initial population size $N_{1}=10$for each simulation. We calculated annual recruitment and survival as $R_{t}= \rho_{t}\times N_{t}$ and $S_{t}= \sigma_{t}\times N_{t}$. We truncated each 30 year simulation at years 10 and 20. Also, for each 10, 20, and 30 year simulation we removed randomly 20% of the population counts and 20% of the annual recruitment data. Therefore, from each 30 year, complete dataset, we ended up with 6 simulated datasets (i.e., 30 years - complete, 30 years – incomplete, 20 years - complete, 20 years – incomplete, 10 years – complete, 10 years – incomplete). We simulated 100 complete, 30 year datasets for each level of *κ*, which resulted in 600 simulated datasets for each level of *κ* and 1800 total simulated datasets.

From each simulated dataset, we input simulated recruitment ($R_{t})$, survival ($S_{t}$), and population counts ${(N}_{t})$or missing data for each year. We used the normal approximation to the binomial to estimate $\rho_{t}$ and $\sigma_{t}$:

$$R_{t} \sim normal\left( \mu_{\rho,t}, \tau_{\rho,t} \right)$$

$$\mu_{\rho,t}= \rho_{t}\times N_{t}$$

$$\tau_{\rho,t}= \rho_{t}\times N_{t}\times(1-\rho_{t})$$

$$S_{t} \sim normal\left( \mu_{\sigma,t}, \tau_{\sigma,t} \right)$$

$$\mu_{\sigma,t}= \sigma_{t}\times N_{t}$$

$$\tau_{\sigma,t}= \sigma_{t}\times N_{t}\times(1-\sigma_{t})$$

Where *t* is the index of years from *1, …, T* number of years (*T* = 10, 20, or 30 years for the length of the simulation). We used vague beta priors on the recruitment and survival rates: $\rho_{t}\sim beta(1, 1)$ and $\sigma_{t}\sim beta(1, 1)$. Our process model for each simulation was:

$$\mu_{t}=N_{t-1}\times(\sigma_{t}+\rho_{t}+\kappa)$$

$$N_{t} \sim normal(\mu_{t},\tau)$$

We fixed the measurement error $\tau= 2^{2}$ and assumed a vague prior on the correction factor, $\kappa\sim uniform\left( -1, 1 \right).$

We sampled the posterior $\hat{\mu}_{\rho1}$ as the mean of $\rho_{t}$ for $t=1, 2, \ldots, 6$ to compare with the simulated value $\mu_{\rho1}$. In a similar fashion, we derived the posteriors $\hat{\mu}_{\rho2}$, $\hat{\mu}_{\sigma1},$and $\hat{\mu}_{\sigma2}$ to compare with the simulated values $\mu_{\rho2}, \mu_{\sigma1}$, and $\mu_{\sigma2}$. We assumed that the model was consistent with the data if the known input parameters were within the 50% credible interval of the posterior estimates at least half the time ([Gelman *et al.*, 2003](#_ENREF_1)). We judged that our model had good power to recover the correction factor if ≥ 50% of the simulations had 50% credible intervals from the posteriors that overlapped the known$\kappa\text{s}$. We left out the last year of simulated data ($N_{11},N_{21,}$ or $N_{31}$) and predicted that value. We judged the model as having good predictive power if the last year of data was within the 50% credible interval of the posterior predictive distribution in at least half of the simulations ([Gelman *et al.*, 2003](#_ENREF_1)). We ran 1 Monte Carlo Markov Chain, discarded the first 500 iterations as burn-in, and ran 1000 more iterations to sample the posteriors with Gibbs sampler. We conducted all analysis with program JAGS through program R.

**Assessment**

Overall, 64.6% and 62.0% of the simulations had 50% prediction intervals for mean recruitment rates that overlapped the simulated values of the recruitment rates (within 50% prediction intervals). Fewer of the simulations (34.3% and 35.4%) had 50% prediction intervals for the mean survival rates that overlapped the simulated values of the survival rates. There was little difference by level of correction factor, years of data, or amount of missing data. We conclude that the fitted model represents the simulated data because approximately half of the simulations predicting the recruitment and survival rates were within their 50% prediction intervals.

The next year’s population size was contained by the 50% prediction interval in 17.6% of the simulations and was contained by the 95% prediction interval in 45.7% of the simulations. This relatively low rate of prediction was primarily because there was very high confidence around the predictions leading to narrow prediction intervals and low coverage (average standard deviation on next year’s population size prediction = 4.27). Again, there was little difference by level of correction factor, years of data, or amount of missing data. Simulations that did not predict the next year’s population size within its 50% prediction interval did not systematically over- or underestimate it, which means our model was not producing biased predictions of next year’s population size.

The known, simulated value of the correction factor (*κ* = 0, -0.05, -0.1) was within the 50% prediction interval for the estimated *κ* in 72.5% of the simulations, and simulations with more years of data had better predictive power. The 50% prediction interval on *κ* did not overlap 0 in 7.2%, 80.0%, and 92.7% of the simulations with *κ* = 0, -0.05, and -0.1, indicating high power to detect a correction factor when present. The median *κ*s from the 18 simulation sets were within 3% of the simulated *κ* in each case, but the accuracy improved greatly with the number of years of data (Fig. S1). Overall, there was a tendency to underestimate *κ*, especially when there were fewer years of data (Fig. S1). Our model was robust to some level of missing data because there was little difference in estimating *κ* from the full dataset or from an incomplete dataset (Fig. S1).

Overall, our simulation study showed that our method recovered the correction factor and other parameters. Prediction was not the strong point of our model. Our model was robust to some level of missing data, and the model estimated the correction factor with higher accuracy and precision when there were more years of data.


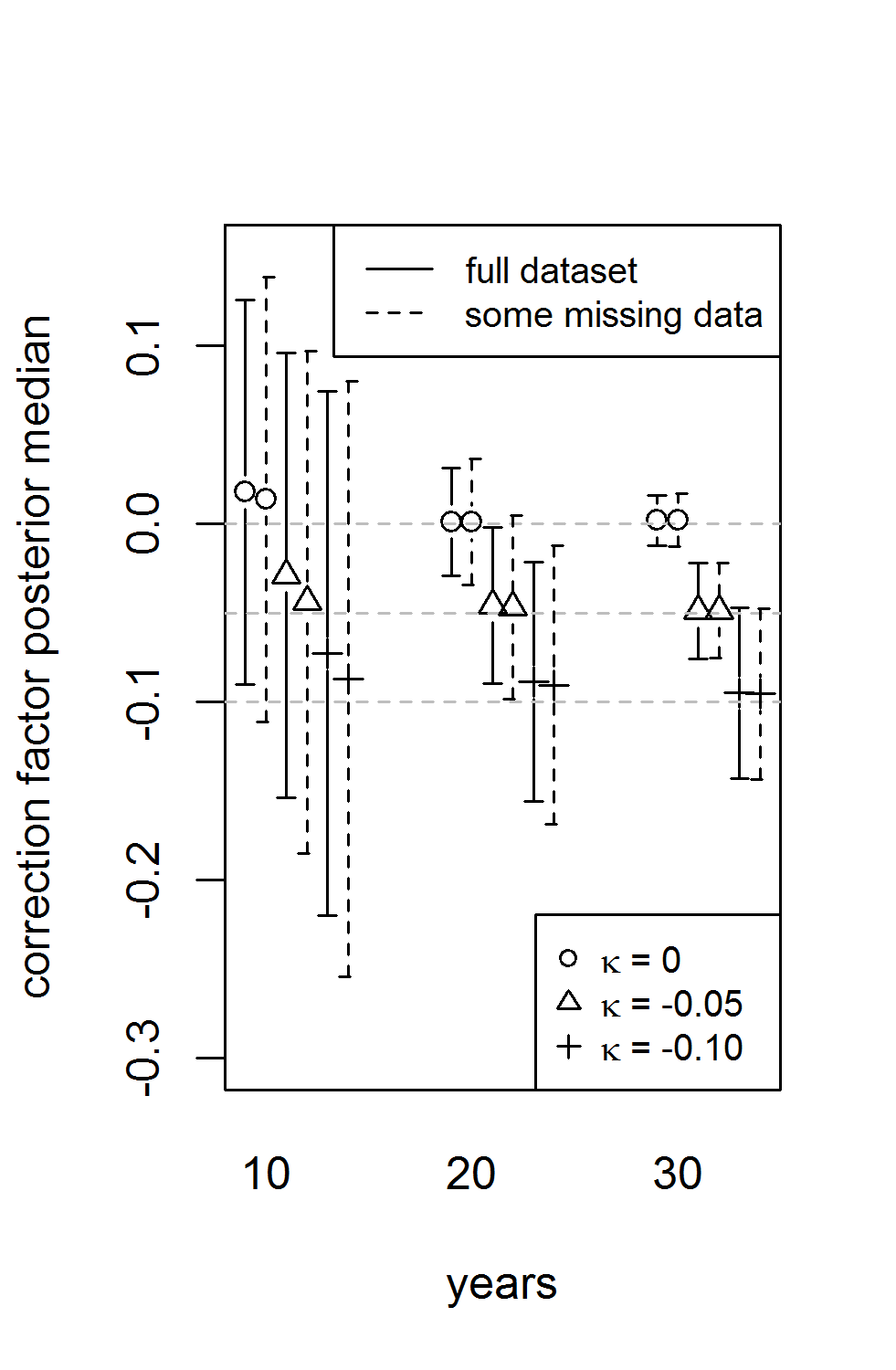


Fig. S1. Median correction factor (*κ*) from 100 simulations and 95% credibility interval for full datasets (solid line) and datasets with 20% missing values (dashed line) using 10, 20, or 30 years of data and a κ of 0, -5%, or -10% per year.

**R code for conducting simulations and models used in simulations**

## set working directory that has 3 model files

## (find models at the end)

## need to install JAGS and R library ‘rjags’, also load package ‘rjags’

# column names for simulation file

output = c("sim","years","full","Npred","kappa","mean.kappa","sd.kappa","low95.kappa","low50.kappa","median.kappa","high50.kappa","high95.kappa","mean.Npred","sd.Npred","low95.Npred","low50.Npred","median.Npred","high50.Npred","high95.Npred","mean.R1","sd.R1","low95.R1","low50.R1","median.R1","high50.R1","high95.R1","mean.R2","sd.R2","low95.R2","low50.R2","median.R2","high50.R2","high95.R2",

"mean.S1","sd.S1","low95.S1","low50.S1","median.S1","high50.S1","high95.S1",

"mean.S2","sd.S2","low95.S2","low50.S2","median.S2","high50.S2","high95.S2")

# simulated dataset

for (i in 0:2) {

kappa = i*0.05

for (k in 1:100) {

r <- NULL

s <- NULL

r[1:6] <- rnorm(6,0.35,0.05)

r[7:31] <- rnorm(25,0.45,0.05)

s[1:18] <- rnorm(18,0.8,0.05)

s[19:31] <- rnorm(13,0.7,0.05)

N <- NULL

N[1] <- 10

mu <- NULL

for (j in 2:31) {

mu[j] <- (N[j-1]*(r[j]+s[j]-kappa))

N[j] <- max(1,rnorm(1,(mu[j]),2))

}

N = round(N)

R = round(r*N)

S = round(s*N)

N.10 = N[1:10]

N.20 = N[1:20]

N.30 = N[1:30]

R.10 = R[1:10]

R.20 = R[1:20]

R.30 = R[1:30]

S.10 = S[1:10]

S.20 = S[1:20]

S.30 = S[1:30]

N.10pred = N[11]

N10 = N[10]

N.20pred = N[21]

N20 = N[20]

N.30pred = N[31]

N30 = N[30]

t1 = round(runif(2,2,5))

t2 = round(runif(2,6,10))

t3 = round(runif(2,11,15))

t4 = round(runif(2,16,20))

t5 = round(runif(2,21,25))

t6 = round(runif(2,26,30))

R[c(t1[1],t2[1],t3[1],t4[1],t5[1],t6[1])]=NA

N[c(t1[2],t2[2],t3[2],t4[2],t5[2],t6[2])]=NA

N.10m = N[1:10]

N.20m = N[1:20]

N.30m = N[1:30]

R.10m = R[1:10]

R.20m = R[1:20]

R.30m = R[1:30]

# run simulations with simulated datasets

params <- c("N.pred","R1","R2","S1","S2","kappa")

model="model.sims.IPM.30yr.txt"

data <- list(N = N, R = R, S = S)

jags <- jags.model(model, data=data, n.chains=2, n.adapt=500)

out <- coda.samples(jags,params,1000)

new = round(c(k,30,1,N.30pred,kappa,mean(out$kappa),sd(out$kappa),quantile(out$kappa,c(.025,.25,.5,.75,.975)),mean(out$N.pred),sd(out$N.pred),quantile(out$N.pred,c(.025,.25,.5,.75,.975)),mean(out$R1),sd(out$R1),quantile(out$R1,c(.025,.25,.5,.75,.975)),mean(out$R2),sd(out$R2),quantile(out$R2,c(.025,.25,.5,.75,.975)),mean(out$S1),sd(out$S1),quantile(out$S1,c(.025,.25,.5,.75,.975)),mean(out$S2),sd(out$S2),quantile(out$S2,c(.025,.25,.5,.75,.975))),4)

output = rbind(output,new)

data <- list(N = N.30m, R = R.30m, S = S.30)

jags <- jags.model(model, data=data, n.chains=1, n.adapt=500)

out <- jags.samples(jags,params,1000)

new = round(c(k,30,0,N.30pred,kappa,mean(out$kappa),sd(out$kappa),quantile(out$kappa,c(.025,.25,.5,.75,.975)),mean(out$N.pred),sd(out$N.pred),quantile(out$N.pred,c(.025,.25,.5,.75,.975)),mean(out$R1),sd(out$R1),quantile(out$R1,c(.025,.25,.5,.75,.975)),mean(out$R2),sd(out$R2),quantile(out$R2,c(.025,.25,.5,.75,.975)),mean(out$S1),sd(out$S1),quantile(out$S1,c(.025,.25,.5,.75,.975)),mean(out$S2),sd(out$S2),quantile(out$S2,c(.025,.25,.5,.75,.975))),4)

output = rbind(output,new)

model="model.sims.IPM.20yr.txt"

data <- list(N = N.20, R = R.20, S = S.20)

jags <- jags.model(model, data=data, n.chains=1, n.adapt=500)

out <- jags.samples(jags,params,1000)

new = round(c(k,20,1,N.20pred,kappa,mean(out$kappa),sd(out$kappa),quantile(out$kappa,c(.025,.25,.5,.75,.975)),mean(out$N.pred),sd(out$N.pred),quantile(out$N.pred,c(.025,.25,.5,.75,.975)),mean(out$R1),sd(out$R1),quantile(out$R1,c(.025,.25,.5,.75,.975)),mean(out$R2),sd(out$R2),quantile(out$R2,c(.025,.25,.5,.75,.975)),mean(out$S1),sd(out$S1),quantile(out$S1,c(.025,.25,.5,.75,.975)),mean(out$S2),sd(out$S2),quantile(out$S2,c(.025,.25,.5,.75,.975))),4)

output = rbind(output,new)

data <- list(N = N.20m, R = R.20m, S = S.20)

jags <- jags.model(model, data=data, n.chains=1, n.adapt=500)

out <- jags.samples(jags,params,1000)

new = round(c(k,20,0,N.20pred,kappa,mean(out$kappa),sd(out$kappa),quantile(out$kappa,c(.025,.25,.5,.75,.975)),mean(out$N.pred),sd(out$N.pred),quantile(out$N.pred,c(.025,.25,.5,.75,.975)),mean(out$R1),sd(out$R1),quantile(out$R1,c(.025,.25,.5,.75,.975)),mean(out$R2),sd(out$R2),quantile(out$R2,c(.025,.25,.5,.75,.975)),mean(out$S1),sd(out$S1),quantile(out$S1,c(.025,.25,.5,.75,.975)),mean(out$S2),sd(out$S2),quantile(out$S2,c(.025,.25,.5,.75,.975))),4)

output = rbind(output,new)

model="model.sims.IPM.10yr.txt"

data <- list(N = N.10, R = R.10, S = S.10)

jags <- jags.model(model, data=data, n.chains=1, n.adapt=500)

out <- jags.samples(jags,params,1000)

new = round(c(k,10,1,N.10pred,kappa,mean(out$kappa),sd(out$kappa),quantile(out$kappa,c(.025,.25,.5,.75,.975)),mean(out$N.pred),sd(out$N.pred),quantile(out$N.pred,c(.025,.25,.5,.75,.975)),mean(out$R1),sd(out$R1),quantile(out$R1,c(.025,.25,.5,.75,.975)),mean(out$R2),sd(out$R2),quantile(out$R2,c(.025,.25,.5,.75,.975)),mean(out$S1),sd(out$S1),quantile(out$S1,c(.025,.25,.5,.75,.975)),mean(out$S2),sd(out$S2),quantile(out$S2,c(.025,.25,.5,.75,.975))),4)

output = rbind(output,new)

data <- list(N = N.10m, R = R.10m, S = S.10)

jags <- jags.model(model, data=data, n.chains=1, n.adapt=500)

out <- jags.samples(jags,params,1000)

new = round(c(k,10,0,N.10pred,kappa,mean(out$kappa),sd(out$kappa),quantile(out$kappa,c(.025,.25,.5,.75,.975)),mean(out$N.pred),sd(out$N.pred),quantile(out$N.pred,c(.025,.25,.5,.75,.975)),mean(out$R1),sd(out$R1),quantile(out$R1,c(.025,.25,.5,.75,.975)),mean(out$R2),sd(out$R2),quantile(out$R2,c(.025,.25,.5,.75,.975)),mean(out$S1),sd(out$S1),quantile(out$S1,c(.025,.25,.5,.75,.975)),mean(out$S2),sd(out$S2),quantile(out$S2,c(.025,.25,.5,.75,.975))),4)

output = rbind(output,new)

}

}

# write simulations to an external file

write.table(output,"out.sims.csv",sep=",")

## import results from external file and make calculations

# estimates of kappa compared to simulated kappa

sims = read.csv("out.sims.csv", header=T)

sims$kappa2.check1 = ifelse((-sims$kappa > sims$low50.kappa) ,1,0)

sims$kappa2.check2 = ifelse((-sims$kappa < sims$high50.kappa) ,1,0)

sims$kappa2.check = sims$kappa2.check1 + sims$kappa2.check2

sims$kappa2.diff0 = ifelse(sims$high50.kappa < 0, 1, 0)

sims$kappa2.check

table(sims$kappa2.check,sims$years)

table(sims$kappa2.check,sims$kappa)

table(sims$kappa2.check,sims$full)

table(sims$kappa2.diff0,sims$years)

table(sims$kappa2.diff0,sims$kappa)

table(sims$kappa2.diff0,sims$full)

# predicting next year’s population size

sims$Npred.check1 = ifelse((sims$Npred > sims$low95.Npred) ,1,0)

sims$Npred.check2 = ifelse((sims$Npred < sims$high95.Npred) ,1,0)

sims$Npred.check = sims$Npred.check1 + sims$Npred.check2

table(sims$Npred.check,sims$kappa,sims$years,sims$full)

sims$Npred2.check1 = ifelse((sims$Npred > sims$low50.Npred) ,1,0)

sims$Npred2.check2 = ifelse((sims$Npred < sims$high50.Npred) ,1,0)

sims$Npred2.check = sims$Npred2.check1 + sims$Npred2.check2

table(sims$Npred2.check,sims$kappa,sims$years,sims$full)

table(sims$Npred2.check,sims$full)

table(sims$Npred2.check,sims$kappa)

table(sims$Npred2.check,sims$years)

sims$Npred.CV = sims$sd.Npred / sims$mean.Npred

tapply(sims$Npred.CV, sims$years, mean)

tapply(sims$Npred.CV, sims$kappa, mean)

tapply(sims$Npred.CV, sims$full, mean)

tapply(sims$sd.Npred, sims$years, mean)

mean(sims$sd.Npred)

# estimating recruitment from simulations versus simulated value

sims$R1a.check1 = ifelse((0.35 > sims$low50.R1) ,1,0)

sims$R1a.check2 = ifelse((0.35 < sims$high50.R1) ,1,0)

sims$R1a.check = sims$R1a.check1 + sims$R1a.check2

table(sims$R1a.check,sims$kappa,sims$years,sims$full)

table(sims$R1a.check,sims$kappa)

table(sims$R1a.check,sims$years)

table(sims$R1a.check,sims$full)

sims$R1.CV = sims$sd.R1 / sims$mean.R1

tapply(sims$R1.CV, sims$years, mean)

sims$R2a.check1 = ifelse((0.45 > sims$low50.R2) ,1,0)

sims$R2a.check2 = ifelse((0.45 < sims$high50.R2) ,1,0)

sims$R2a.check = sims$R2a.check1 + sims$R2a.check2

table(sims$R2a.check,sims$kappa,sims$years,sims$full)

table(sims$R2a.check,sims$kappa)

table(sims$R2a.check,sims$years)

table(sims$R2a.check,sims$full)

# estimating survival from simulations versus simulated value

sims$S1a.check1 = ifelse((0.8 > sims$low50.S1) ,1,0)

sims$S1a.check2 = ifelse((0.8 < sims$high50.S1) ,1,0)

sims$S1a.check = sims$S1a.check1 + sims$S1a.check2

table(sims$S1a.check,sims$kappa,sims$years,sims$full)

table(sims$S1a.check,sims$kappa)

table(sims$S1a.check,sims$years)

table(sims$S1a.check,sims$full)

sims$S2a.check1 = ifelse((0.7 > sims$low50.S2) ,1,0)

sims$S2a.check2 = ifelse((0.7 < sims$high50.S2) ,1,0)

sims$S2a.check = sims$S2a.check1 + sims$S2a.check2

table(sims$S2a.check,sims$kappa,sims$years,sims$full)

table(sims$S2a.check,sims$kappa)

table(sims$S2a.check,sims$years)

table(sims$S2a.check,sims$full)

#### Model files

## “model.sims.IPM.30yr.txt”

model {

# recruitment

for (t in 1:30) {

rmean[t] <- rho[t]*N[t]

rtau[t] <- (1/(rho[t]*N[t]*(1-rho[t])))

R[t] ~ dnorm(rmean[t],rtau[t])

}

# survival

for (t in 1:30) {

smean[t] <- sigma[t]*N[t]

stau[t] <- (1/(sigma[t]*N[t]*(1-sigma[t])))

S[t] ~ dnorm(smean[t],stau[t])

}

for (t in 2:30) {

mu[t] <- N[t-1]*(sigma[t] + rho[t] + kappa)

N[t] ~ dnorm(mu[t], tau)

}

# priors

for (i in 1:30) {

rho[i] ~ dbeta(1,1)

sigma[i] ~ dbeta(1,1)

}

kappa ~ dunif(-1,1)

tau <- 0.25

# derived

R1 <- mean(rho[1:6])

R2 <- mean(rho[7:30])

S1 <- mean(sigma[1:18])

S2 <- mean(sigma[19:30])

N.pred <- N[30]*(S2+R2+kappa)

mu.pred ~ dnorm(N.pred,tau)

}

## “model.sims.IPM.20yr.txt”

model {

# recruitment

for (t in 1:20) {

rmean[t] <- rho[t]*N[t]

rtau[t] <- (1/(rho[t]*N[t]*(1-rho[t])))

R[t] ~ dnorm(rmean[t],rtau[t])

}

# survival

for (t in 1:20) {

smean[t] <- sigma[t]*N[t]

stau[t] <- (1/(sigma[t]*N[t]*(1-sigma[t])))

S[t] ~ dnorm(smean[t],stau[t])

}

for (t in 2:20) {

mu[t] <- N[t-1]*(sigma[t] + rho[t] + kappa)

N[t] ~ dnorm(mu[t], tau)

}

# priors

for (i in 1:20) {

rho[i] ~ dbeta(1,1)

sigma[i] ~ dbeta(1,1)

}

kappa ~ dunif(-1,1)

tau <- 0.25

# derived

R1 <- mean(rho[1:6])

R2 <- mean(rho[7:20])

S1 <- mean(sigma[1:18])

S2 <- mean(sigma[19:20])

N.pred <- N[20]*(S2+R2+kappa)

}

## “model.sims.IPM.10yr.txt”

model {

# recruitment

for (t in 1:10) {

rmean[t] <- rho[t]*N[t]

rtau[t] <- (1/(rho[t]*N[t]*(1-rho[t])))

R[t] ~ dnorm(rmean[t],rtau[t])

}

# survival

for (t in 1:10) {

smean[t] <- sigma[t]*N[t]

stau[t] <- (1/(sigma[t]*N[t]*(1-sigma[t])))

S[t] ~ dnorm(smean[t],stau[t])

}

for (t in 2:10) {

mu[t] <- N[t-1]*(sigma[t] + rho[t] + kappa)

N[t] ~ dnorm(mu[t], tau)

}

# priors

for (i in 1:10) {

rho[i] ~ dbeta(1,1)

sigma[i] ~ dbeta(1,1)

}

kappa ~ dunif(-1,1)

tau <- 0.25

# derived

R1 <- mean(rho[1:6])

R2 <- mean(rho[7:10])

S1 <- mean(sigma[1:10])

S2 <- 0

N.pred <- N[10]*(S1+R2+kappa)

}

**References**

Gelman, A., Carlin, J.B., Stern, H.S. & Rubin, D.B. (2003) *Bayesian data analysis*. CRC press.

Wydeven, A.P., Wiedenhoeft, J.E., Schultz, R.N., Thiel, R.P., Jurewicz, R.L., Kohn, B.E. & Van Deelen, T.R. (2009) History, population growth, and management of wolves in Wisconsin. *Recovery of Gray Wolves in the Great Lakes Region of the United States: An Endangered Species Success Story* (ed. by A.P. Wydeven, T.R. Van Deelen and E.J. Heske), pp. 87-105. Springer, New York, NY, USA.
