# Supplementary material for: Are the numbers adding up? Exploiting discrepancies among complementary population models
Source: Ecol Evol. 2014 Dec 24;5(2):368–76. doi: 10.1002/ece3.1365 (PMC4314269; doi:10.1002/ece3.1365)
Supplement: Supplementary file 2 [file ece30005-0368-sd2.docx]

**Appendix S2.** Model assessment

We conducted a thorough model assessment to understand the behavior and performance of an integrated population model (IPM) for population dynamics of Wisconsin wolves. The purpose of the model was to estimate a correction factor that acts as a discrepancy between the estimates that the two data types would have provided if used independently. Here, we provide an empirical justification of our IPM for estimating a correction factor, model checking with external validation, and posterior predictive checking to establish whether our model is consistent with our data. Finally, we conducted a sensitivity analysis to determine whether our model was sensitive to our choice of prior distributions and model for the population count.

*Empirical justification of an IPM for estimating a correction factor*

We provide an empirical justification of the IPM estimating a correction factor as a discrepancy between the estimates of the different data types (recruitment and survival data; population count data). Our justification is based on whether the parameter estimates for survival and recruitment from the IPM are the same as the parameter estimates of survival and recruitment in an unintegrated model. When we run the survival model and the recruitment model by themselves (and separate from each other) and not as part of the IPM we would expect the parameter estimates to be the same as the parameter estimates from the IPM. This finding would demonstrate that the correction factor that we are estimating is actually capturing that discrepancy between the estimates of the different data types. If we do not find this correspondence in the parameter estimates between the IPM and the unintegrated models, then we would conclude that our correction factor was not acting as a discrepancy between the estimates of the different data types.

We ran the unintegrated model and compared the results for recruitment and survival to the integrated model. First, we ran the recruitment portion of the model by itself (without the survival information or population count information) and plotted the annual posterior means in red in Figure S2.1. Next, we ran the unintegrated survival model by itself and plotted the posterior mean in red with a dotted line in Figure S2.1. The posterior estimates from our integrated model are essentially identical to the posterior estimates from our unintegrated model (Figure S2.1). This provides the empirical justification for establishing our correction factor as a discrepancy between the estimates. If these two data types were used separately, we would have gotten the same estimates for recruitment and survival as we got in the integrated model.


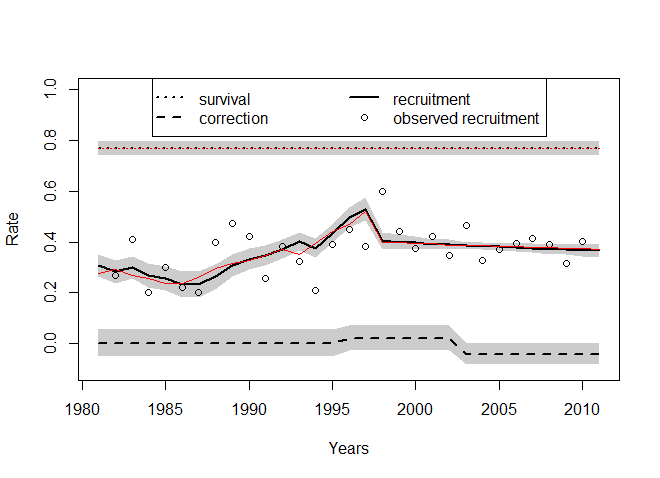


Figure S2.1. Observed recruitment and posterior estimates and 95% credibility intervals for annual recruitment, survival, and an estimated correction factor in the Wisconsin, USA wolf population from 1980 – 2012 (Figure 3 from main text). The red line is the mean posterior estimates of recruitment for each year when recruitment was run alone, and the red dotted line is the mean posterior estimate of the survival rate when the survival data are run alone.

*External validation*

We implemented an external validation for our IPM. The validation data that we left out of the model was the population size in 2012. We predicted the 2012 population size from the posterior distribution:

$$N_{2012}\sim lognormal\left( \mu_{2012}, \upsilon^{2} \right)$$

$$\mu_{2012}=log(N_{2011}\times\left( \rho_{2012}+\sigma_{2012}+\kappa_{2012} \right))$$

We concluded that if the mean of the posterior $N_{2012}$ was contained within the estimated 2012 population size of 815 – 880 wolves, then our model provided reasonable inference.

The mean of the posterior $N_{2012}$ = 878 which was within the estimated 2012 population size of 815 – 880 wolves. Therefore, we concluded that the inferences from our model made sense.

*Posterior predictive checking*

We used a graphical approach to the posterior predictive check and looked for discrepancies between the real and simulated data ([Gelman *et al.*, 2003](#_ENREF_1)). We plotted the exponential of the mean of the posteriors $\mu_{t}$ for $t=2, 3, \ldots32$along with the observed mean wolf population counts from Wisconsin from 1981 – 2011. We assessed whether the simulated data matched the observed data and whether there were any systematic discrepancies in the simulated data compared to the real data. If in the graphical assessment we saw close correspondence between the simulated and real datasets, then we determined that the data fit the model well. To look for normality in the residuals in the population count model, we plotted (observed – expected) versus expected and looked for patterns in the residuals as evidence for poor model fit. The observed data were the posterior means of $\mu_{t}$ for $t=2, 3, \ldots32$ and the expected data were the log population counts from Wisconsin from 1981 – 2011.

We plotted the exponential of the mean of the posteriors (red line, Fig. S2.2) along with the observed population counts in 1981 – 2011 (open circles, Fig. S2.2) and found close correspondence. Also, we plotted the residuals (the difference between the mean of the posteriors and the log observed population plotted against the log observed population value) and found that there was no pattern in the residuals (Fig. S2.3).


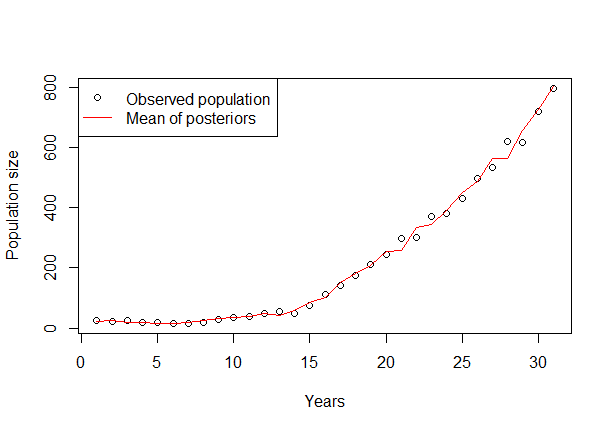


Figure S2.2. Wisconsin wolf population size from 1981 – 2011 as estimated from the means of the posteriors in an IPM compared to what was observed.


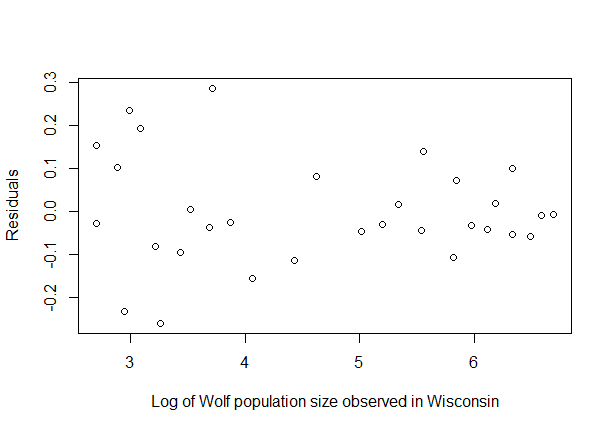


Figure S2.3. Residual plot (observed – expected versus expected) for population count data from an IPM of wolf population dynamics from Wisconsin, USA.

*Sensitivity analysis*

We ran a sensitivity analysis on our choice of prior parameters in the survival model, the distribution of population counts, and the prior distribution on the correction factor. In the survival portion of the IPM, we chose a value of $r=0.01$, which corresponds to our prior guess at the failure rate, and $c=0.001$, which corresponds to our confidence in our guess about the failure rate (lower values of $c$ mean a lower confidence in our guess). In a one-factor-at-a-time sensitivity analysis, we varied the values of $c=\{0.1, 0.01,0.0001\}$ and $r=\{0.05, 0.2\}$ in the IPM and assessed whether the posterior estimates for survival were affected by our prior choice of $r$ and $c$. To explore whether our choice of prior for the correction factor affected the posterior estimates, we ran the model with $\kappa\sim uniform(0,1)$ which would assume that the correction factor was always an addition to the population, and we ran the model with $\kappa\sim uniform(-1,0)$ to assume that the correction factor was always a subtraction from the population. Also, we ran the IPM with a Poisson distribution for the population counts (instead of lognormal). In the sensitivity analysis for the correction factor and population counts, we assessed whether our choice of distribution led to a change in the posterior parameter estimates.

For the survival portion of the sensitivity analysis, we found that the mean and SD of the posterior survival rate were robust to our choice of $r$ and $c$ (Table S2.1). When we used a Poisson distribution for the population counts, we found that the mean parameter estimates were very similar to the original model (lognormal distribution for population counts), except for an increase in variance on the correction factors (Fig. S2.4). A change in the prior on the correction factor to a Uniform(0,1) had the effect of reducing the mean survival rate and increasing the mean correction factor compared to the original model (Fig. S2.5). A change in the prior on the correction factor to a Uniform(-1,0) had the effect of increasing the mean survival rate and decreasing the mean correction factor compared to the original model (Fig. S2.6). These results were not surprising and we highlight our use of a Uniform(-1,1) for the correction factor in our original model so that we are not making an unfounded assumption about the correction being a net addition or subtraction from the population.

Table S2.1. Posterior mean and standard deviation (SD) of annual wolf survival rate from radio-collared wolves in Wisconsin, USA, under different prior values of r and c (parameters used in the construction of the survival rate priors).

| Value of $r$ | Value of $c$ | Mean of posterior | SD of posterior |
| --- | --- | --- | --- |
| 0.1 | 0.001 | 0.7688 | 0.0141 |
| 0.1 | 0.0001 | 0.7688 | 0.0141 |
| 0.1 | 0.01 | 0.7686 | 0.0144 |
| 0.1 | 0.1 | 0.7652 | 0.0140 |
| 0.05 | 0.001 | 0.7688 | 0.0139 |
| 0.2 | 0.001 | 0.7686 | 0.0141 |


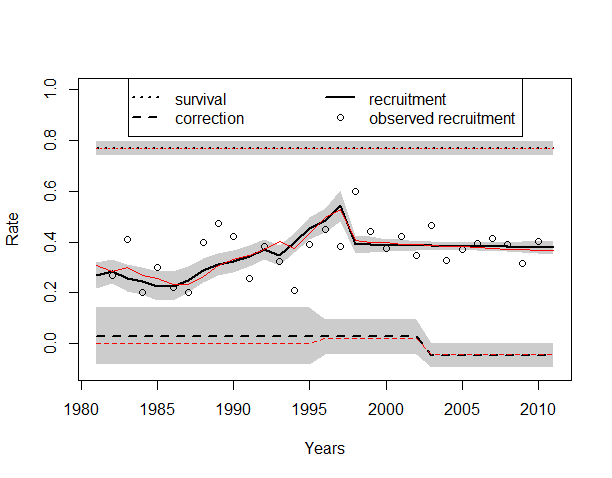
Figure S2.4. Observed recruitment and posterior estimates and 95% credibility intervals for annual recruitment, survival, and an estimated correction factor in the Wisconsin, USA wolf population from 1980 – 2012 when population counts were modeled with a **Poisson distribution**. The red lines (solid, dashed, and dotted) are the corresponding mean posterior estimates from the original model (Figure 3 of main text).


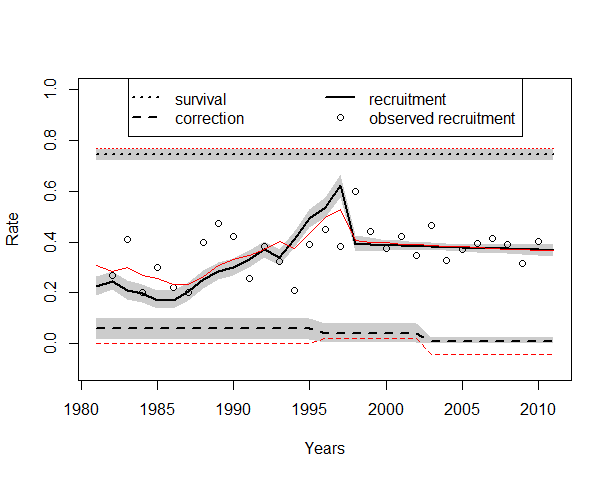


Figure S2.5. Observed recruitment and posterior estimates and 95% credibility intervals for annual recruitment, survival, and an estimated correction factor in the Wisconsin, USA wolf population from 1980 – 2012 when the prior on the correction factor was restricted to **Uniform(0,1)**. The red lines (solid, dashed, and dotted) are the corresponding mean posterior estimates from the original model (Figure 3 of main text).


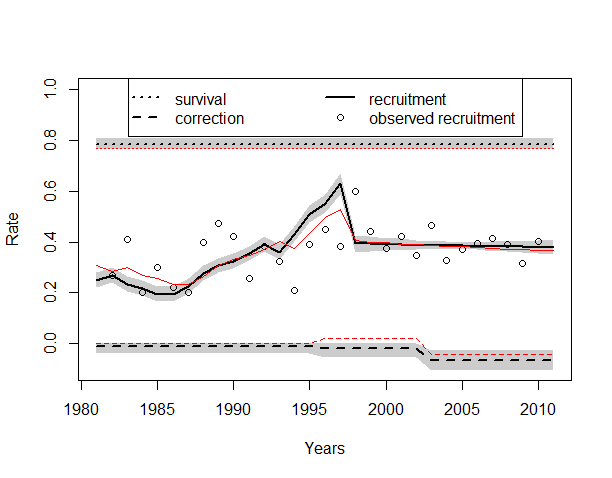


Figure S2.6. Observed recruitment and posterior estimates and 95% credibility intervals for annual recruitment, survival, and an estimated correction factor in the Wisconsin, USA wolf population from 1980 – 2012 when the prior on the correction factor was restricted to **Uniform(-1,0)**. The red lines (solid, dashed, and dotted) are the corresponding mean posterior estimates from the original model (Figure 3 of main text).

**References**

Gelman, A., Carlin, J.B., Stern, H.S. & Rubin, D.B. (2003) *Bayesian data analysis*. CRC press.
