# Supplementary material for: Are the numbers adding up? Exploiting discrepancies among complementary population models
Source: Ecol Evol. 2014 Dec 24;5(2):368–76. doi: 10.1002/ece3.1365 (PMC4314269; doi:10.1002/ece3.1365)
Supplement: Supplementary file 3 [file ece30005-0368-sd3.docx]

Appendix S3. Bayesian integrated population model with correction factor for use with JAGS and survival data

#### example of an integrated population model using

#### recruitment, radio-telemetry survival data and population

#### count data to estimate 3 correction factors

### recruitment is modeled with a change-point model that varies

### with log of standardized population size.

### survival does not vary with population size

## survival data

# Notation and general survival model follows WinBUGS example

# “Leuk: survival analysis using Cox regression”

# N.surv = the number of survival records

# T = the number of unique end points during the year

# eps = very small value (e.g., 0.000001) to help numerical

# precision in step function

# obs.t = end time for all records; see end for data

# start.t = start time for all records; see end for data

# fail = censoring indicator (fail = 1 for death, fail = 0

# otherwise) for all records, see end for data

## recruitment and population data

# P = number of new recruits

# N = population size

# tau.proc = precision of annual population counts

# log.N.standard = log of standardized population size

# changepoint = year that change-point model changes

### setup survival data

data {

for(i in 1:N.surv) {

for(j in 1:T) {

Y[i,j] <- step(obs.t[i] - t[j] + eps)*step(t[j] - start.t[i] + eps) # risk set = 1 if obs.t >= t and start.t <= t

dN[i, j] <- Y[i, j] * step(t[j + 1] - obs.t[i] - eps) * fail[i]

# counting process jump if obs.t is in interval and fail = 1

}

}

}

# Integrated population model

model {

# survival

for(j in 1:T) {

for(i in 1:N.surv) {

dN[i, j] ~ dpois(Idt[i, j])

Idt[i, j] <- Y[i, j] * dL0[j] # Intensity process

}

dL0[j] ~ dgamma(mu2[j], c)

mu2[j] <- dL0.star[j] * c

S[j] <- exp(-sum(dL0[1 : j]))

}

c <- 0.001 # confidence in guess for dL0

r <- 0.1 # prior guess at failure rate

for (j in 1 : T) { dL0.star[j] <- r * (t[j + 1] - t[j]) }

# prior guess at the hazard function

S.annual <- S[T]

# recruitment

for (t in 2:changepoint) {

mean[t] <- rho[t]*N[t-1]

tau[t] <- (1/(rho[t]*N[t-1]*(1-rho[t])))

P[t] ~ dnorm(mean[t],tau[t])

logit(rho[t]) <- beta.rho1[1] + beta.rho1[2]*log.N.standard[t-1]

}

for (t in (changepoint+1):years) {

mean[t] <- rho[t]*N[t-1]

tau[t] <- (1/(rho[t]*N[t-1]*(1-rho[t])))

P[t] ~ dnorm(mean[t],tau[t])

logit(rho[t]) <- beta.rho2[1] + beta.rho2[2]*log.N.standard[t-1]

}

for (i in 1:2) {

beta.rho1[i] ~ dnorm(0,0.0001)

beta.rho2[i] ~ dnorm(0,0.0001)

}

# population

for (i in 2:16) {

mu[i] <- max(log(2),log(N[i-1]*(rho[i] + S.annual + correction[1])))

N[i] ~ dlnorm(mu[i],tau.proc)

}

for (i in 17:23) {

mu[i] <- max(log(2),log(N[i-1]*(rho[i] + S.annual + correction[2])))

N[i] ~ dlnorm(mu[i],tau.proc)

}

for (i in 24:years) {

mu[i] <- max(log(2),log(N[i-1]*(rho[i] + S.annual + correction[3])))

N[i] ~ dlnorm(mu[i],tau.proc)

}

for (i in 1:3) {

correction[i] ~ dunif(-1,1)

}}

### survival data: start.t, obs.t, fail

### format for use in R with model, above

start.t = c(276,285,130,1,136,1,143,1,1,147,1,149,201,1,164,29,1,1,1,1,85,1,326,1,1,111,1,1,1,1,248,1,1,1,1,312,1,305,1,1,352,1,1,1,1,1,27,48,1,1,73,1,1,40,1,1,1,313,1,95,268,1,28,114,86,326,1,1,1,1,156,1,1,1,1,1,41,1,133,130,1,1,135,1,136,139,1,140,281,1,134,134,142,1,142,1,144,224,1,132,1,138,1,133,1,1,209,1,135,1,130,132,133,146,133,1,1,1,1,1,145,1,140,1,1,1,1,204,1,1,206,1,1,145,126,1,286,1,1,1,1,136,1,208,1,203,1,1,201,1,1,1,1,1,1,221,1,142,1,1,1,1,218,1,1,141,134,194,1,195,1,200,204,1,1,1,1,1,134,1,1,233,1,1,234,1,1,158,1,129,1,1,137,1,1,158,1,201,126,1,1,1,1,179,187,195,196,132,124,1,1,240,1,150,125,174,1,1,1,160,211,1,1,1,132,1,1,1,141,1,210,1,1,1,1,167,1,1,1,131,1,1,129,209,1,144,162,1,296,1,1,1,1,211,1,219,1,1,220,1,1,1,241,1,152,1,1,1,139,1,184,1,127,1,1,253,1,1,1,1,145,1,1,1,1,1,176,1,190,1,1,1,1,1,1,1,1,248,1,1,1,1,133,1,1,133,133,141,1,1,248,248,124,1,1,1,1,141,1,1,157,172,1,1,324,1,1,1,1,1,1,179,1,195,204,1,1,1,1,211,1,1,1,1,101,1,1,1,1,1,180,1,1,1,1,1,298,253,1,1,143,1,162,163,1,1,236,1,246,1,1,304,1,157,1,165,1,1,197,1,1,240,197,1,1,207,1,147,220,1,1,1,1,241,1,1,1,1,1,241,1,1,1,1,1,1,1,1,1,260,1,1,241,1,1,132,1,1,143,1,161,1,250,1,133,1,133,1,1,102,130,1,1,1,235,1,236,1,1,1,1,1,248,1,1,1,1,301,142,156,1,1,1,133,178,1,1,1,1,141,1,1,1,143,1,1,143,1,1,145,1,1,1,148,1,1,1,153,1,1,1,1,159,1,1,1,181,138,1,262,266,1,319,1,1,1,1,259,264,1,1,1,1,1,1,118,211,247,1,293,1,1,1,132,1,131,1,1,1,144,144,154,1,1,1,199,1,209,1,1,1,1,1,217,1,1,1,151,1,1,1,228,163,1,1,143,169,1,1,1,256,1,179,1,1,1,1,146,1,193,176,1,1,181,1,1,163,1,1,1,206,1,1,1,212,1,1,1,1,217,1,254,1,1,208,1,209,1,215,1,222,1,229,1,204,217,1,1,1,1,1,252,223,225,183,1,1,187,154,1,1,138,152,1,206,1,149,1,207,1,194,141,1,1,142,1,1,1,1,159,1,184,187,1,204,1,176,1,1,1,1,164,1,1,1,1,1,1,172,1,1,1,140,1,199,1,1,201,1,203,1,1,1,200,1,1,1,1,1,199,193,1,1,1,253,1,247,1,299,1,290,1,152,1,1,136,347,1,1,183,1,1,218,1,247,311,1,180,1,1,1,1,1,138,1,1,1,176,1,1,1,1,1,1,172,176,1,1,1,1,247,1,235,1,241,1,188,1,1,161,167,237,1,1,250,1,1,1,249,1,1,1,1,235,176,316,1,179,1,1,1,1,253,1,1,59,160,1,1,1,1,1,167,1,1,134,1,1,1,147,1,1,1,1,1,1,1,287,1,1,1,238,233,1,233,1,176,1,151,1,1,194,1,1,1,1,1,1,1,1,1,300,1,77,1,1,179,1,1,1,1,251,1,1,1,217,1,136,1,1,1,151,154,164,1,1,1,1,305,1,1,1,147,1,144,1,1,144,1,172,222,1,1,138,271,1,138,164,1,1,1,1,146,1,1,176,1,1,1,224,1,1,1,177,158,1,1,162,239,1,139,1,1,1,1,1,125,1,131,171,168,166,1,1,133,1,1,1,270,1,144,1,1,1,177,1,1,1,1,1,162,1,142,1,364,1,156,1,131,1,1,1,1,1,1,138,144,1,1,134,122,1,175,1,154,1,194,1,270,155,1,161,1,1,1,1,1,135,1,1,1,1,1,302,1,1,129,1,147,139,164,149,1,1,1,1,152,178,1,1,178,1,1,1,1,1,179,1,1,1,169,1,1,1,1,168,1,1,136,296,1,168,1,1,324,1,1,1,1,182,1,1,1,293,1,168,301,1,162,130,145,153,1,182,1,1,365,1,1,157,1,1,1,1,172,1,1,1,228,1,1,1,1,250,159,1,1,1,51,168,1,314,1,1,1,156,1,202,315,247,1,174,1,307,1,1,1,313,1,1,1,1,150,1,1,156,1,1,228,1,1,1,179,117,1,1,1,171,1,1,1,173,1,1,182,1,1,1,1,301,1,250,1,169,158,168,1,180,36,138,1,265,150,1,1,181,175,1,1,1,180,1,1,1,1,301,1,1,322,1,1,304,1,1,1,171,1,1,192,1,1,180,1,180,1,1,313,313,300,1,236,1,148,1,1,1,1,1,1,64,176,1,1,291,1,1,179,1,142,1,1,1,306,1,302,1,1,1,126,1,1,1,127,1,1,1,307,1,1,1,158,154,1,171,1,319,1,320,1,131,1,1,132,265,255,1,139,1,1,1,176,1,158,181,1,1,1,243,210,1,212,1,1,1,272,195,161,1,306,181,1,132,1,42,291,355,1,1,294,1,1,207,298,1,1,1,316,1,179,1,178,1,2,137,134,1,153,176,1,165,1,42,259,1,1,194,194,1,300,312,1,107,1,311,1,170,1,167,1,1,27,159,1,130,107,1,312,1,1,135)

obs.t = c(333,327,365,48,365,204,365,365,341,365,239,266,365,209,230,365,365,365,365,194,365,5,365,365,149,365,365,365,365,4,365,365,365,365,161,365,17,365,365,326,365,365,365,365,365,178,35,365,365,142,365,365,39,365,365,365,71,365,190,334,365,79,122,125,99,365,365,365,365,301,365,365,365,365,365,273,365,325,344,365,365,327,365,26,208,365,363,251,365,341,299,318,365,87,365,39,347,365,174,365,153,365,59,365,365,274,365,11,365,35,350,214,250,229,365,365,365,365,365,135,365,14,365,365,365,365,135,365,365,55,365,365,29,334,365,11,365,365,365,365,45,365,170,365,69,365,365,341,365,365,365,365,365,365,340,365,29,365,365,365,365,239,365,365,102,313,313,365,38,365,16,282,365,365,365,365,365,107,365,365,26,365,365,27,365,365,120,365,236,365,365,211,365,365,353,365,183,361,365,365,365,365,239,267,344,220,256,289,365,365,271,365,76,246,313,365,365,365,79,331,365,365,365,206,365,365,365,3,365,182,365,365,365,365,44,365,365,365,142,365,365,240,306,365,236,166,365,250,365,365,365,365,72,365,13,365,365,346,365,365,365,118,365,346,365,365,365,310,365,91,365,76,365,365,149,365,365,365,365,66,365,365,365,365,365,41,365,263,365,365,365,365,365,365,365,365,45,365,365,365,365,217,365,365,52,235,336,365,365,10,353,353,365,365,365,365,244,365,365,121,354,365,365,202,365,365,365,365,365,365,218,365,344,351,365,365,365,365,163,365,365,365,365,324,365,365,365,365,365,205,365,365,365,365,365,206,344,365,365,30,365,24,354,365,365,267,365,60,365,365,118,365,82,365,14,365,365,70,365,365,198,337,365,365,98,365,21,308,365,365,365,365,304,365,365,365,365,365,92,365,365,365,365,365,365,365,365,365,4,365,365,323,365,365,266,365,365,165,365,17,365,92,365,155,365,144,365,365,361,247,365,365,365,45,365,130,365,365,365,365,365,76,365,365,365,365,38,313,281,365,365,365,305,308,365,365,365,365,224,365,365,365,149,365,365,45,365,365,284,365,365,365,175,365,365,365,140,365,365,365,365,6,365,365,365,224,231,365,331,319,365,88,365,365,365,365,195,319,365,365,365,365,365,365,23,136,317,365,110,365,365,365,8,365,145,365,365,365,329,213,363,365,365,365,323,365,10,365,365,365,365,365,303,365,365,365,9,365,365,365,9,323,365,365,64,323,365,365,365,353,365,73,365,365,365,365,145,365,2,290,365,365,78,365,365,119,365,365,365,174,365,365,365,313,365,365,365,365,213,365,16,365,365,13,365,15,365,15,365,15,365,15,365,15,329,365,365,365,365,365,95,310,280,284,365,365,245,343,365,365,117,184,365,228,365,295,365,69,365,44,273,365,365,34,365,365,365,365,135,365,13,336,365,64,365,7,365,365,365,365,179,365,365,365,365,365,365,10,365,365,365,354,365,356,365,365,192,365,76,365,365,365,61,365,365,365,365,365,183,273,365,365,365,27,365,345,365,353,365,97,365,98,365,365,166,351,365,365,308,365,365,11,365,118,357,365,234,365,365,365,365,365,204,365,365,365,193,365,365,365,365,365,365,287,237,365,365,365,365,28,365,18,365,286,365,56,365,365,216,318,343,365,365,335,365,365,365,240,365,365,365,365,244,329,279,365,44,365,365,365,365,169,365,365,52,76,365,365,365,365,365,12,365,365,333,365,365,365,228,365,365,365,365,365,365,365,100,365,365,365,324,357,365,74,365,74,365,4,365,365,123,365,365,365,365,365,365,365,365,365,84,365,73,365,365,108,365,365,365,365,20,365,365,365,73,365,353,365,365,365,301,363,321,365,365,365,365,201,365,365,365,129,365,324,365,365,131,365,24,321,365,365,38,305,365,17,329,365,365,365,365,99,365,365,253,365,365,365,248,365,365,365,364,245,365,365,283,343,365,40,365,365,365,365,365,191,365,190,324,324,324,365,365,42,365,365,365,242,365,18,365,365,365,47,365,365,365,365,365,179,365,178,365,50,365,361,365,303,365,365,365,365,365,365,102,324,365,365,9,138,365,114,365,113,365,275,365,85,353,365,325,365,365,365,365,365,324,365,365,365,365,365,215,365,365,273,365,231,276,262,248,365,365,365,365,271,344,365,365,5,365,365,365,365,365,30,365,365,365,229,365,365,365,365,99,365,365,81,325,365,79,365,365,123,365,365,365,365,347,365,365,365,59,365,263,351,365,350,283,189,325,365,293,365,365,4,365,365,71,365,365,365,365,4,365,365,365,257,365,365,365,365,314,325,365,365,365,236,57,365,13,365,365,365,69,365,305,298,330,365,55,365,356,365,365,365,89,365,365,365,365,219,365,365,100,365,365,99,365,365,365,115,282,365,365,365,331,365,365,365,234,365,365,321,365,365,365,365,255,365,20,365,293,341,317,365,35,314,278,365,214,322,365,365,110,334,365,365,365,51,365,365,365,365,59,365,365,67,365,365,236,365,365,365,100,365,365,87,365,365,325,365,5,365,365,164,334,321,365,144,365,53,365,365,365,365,365,365,21,165,365,365,142,365,365,10,365,108,365,365,365,100,365,10,365,365,365,317,365,365,365,99,365,365,365,100,365,365,365,35,229,365,332,365,33,365,184,365,262,365,365,99,297,327,365,32,365,365,365,100,365,40,164,365,365,365,100,301,365,137,365,365,365,84,333,327,365,59,333,365,38,365,93,51,325,365,365,46,365,365,93,283,365,365,365,100,365,129,365,108,365,79,93,248,365,32,324,365,7,365,45,222,365,365,93,305,365,253,332,365,24,365,100,365,79,365,100,365,365,84,262,365,100,336,365,99,365,365,39,354)

fail = c(0,1,0,1,0,0,0,0,1,0,0,1,0,0,0,0,0,0,0,0,0,1,0,0,0,0,0,0,0,0,0,0,0,0,0,0,0,0,0,1,0,0,0,0,0,0,1,0,0,1,0,0,0,0,0,0,0,0,1,0,0,0,1,1,0,0,0,0,0,0,0,0,0,0,0,0,0,1,1,0,0,1,0,0,1,0,1,0,0,0,1,1,0,1,0,0,0,0,0,0,0,0,0,0,0,0,0,0,0,1,1,0,0,1,0,0,0,0,0,0,0,0,0,0,0,0,0,0,0,1,0,0,0,0,0,0,0,0,0,0,0,0,0,0,0,0,0,0,0,0,0,0,0,0,1,0,0,0,0,0,0,1,0,0,0,0,0,0,1,0,0,1,0,0,0,0,0,0,0,0,1,0,0,1,0,0,0,0,1,0,0,1,0,0,0,0,1,0,0,0,0,0,0,1,0,1,0,0,0,0,0,0,0,1,0,0,0,0,0,0,0,0,0,0,0,0,0,1,0,0,0,0,0,0,0,0,0,0,0,0,0,1,0,0,0,0,0,0,0,0,0,0,0,0,1,0,0,0,0,0,0,0,0,1,0,0,0,1,0,0,0,0,0,0,1,0,0,0,0,0,0,0,0,0,0,0,0,1,0,0,0,0,0,0,0,0,1,0,0,0,0,0,0,0,0,0,1,0,0,1,1,0,0,0,0,0,0,0,0,0,1,0,0,1,0,0,0,0,0,0,1,0,1,1,0,0,0,0,0,0,0,0,0,0,0,0,0,0,0,0,0,0,0,0,0,0,0,0,0,0,0,1,1,0,0,0,0,0,0,0,1,0,0,0,0,0,0,1,0,0,1,1,0,0,1,0,0,0,0,0,0,0,1,0,0,0,0,0,0,0,0,0,0,0,0,0,0,0,1,0,0,1,0,0,0,0,0,0,0,1,0,0,0,0,0,0,0,0,1,0,0,0,0,1,0,0,0,0,0,0,0,0,0,0,0,0,1,0,1,0,0,0,0,0,0,0,0,0,0,0,0,0,1,0,0,1,0,0,1,0,0,0,0,0,0,0,1,0,0,0,0,1,0,0,0,0,1,0,1,0,0,0,0,0,0,0,0,0,0,0,0,0,0,0,1,1,0,0,1,0,0,0,1,0,0,0,0,0,0,1,1,0,0,0,0,0,0,0,0,0,0,0,0,0,0,0,0,0,0,0,0,1,0,0,0,1,0,0,0,1,0,0,0,0,0,0,0,0,1,0,0,0,1,0,0,1,0,0,0,0,0,0,0,0,0,0,0,0,1,0,1,0,0,1,0,0,0,0,0,0,0,0,0,0,1,0,0,0,0,0,0,0,0,0,0,0,1,0,0,0,1,1,0,1,0,0,0,0,0,0,0,0,0,1,0,0,0,0,0,0,0,1,0,0,0,1,0,0,0,0,0,0,0,0,0,0,0,1,0,0,0,1,0,0,0,0,1,0,0,0,0,0,1,0,0,0,0,0,1,0,0,0,0,0,0,0,0,1,0,0,0,0,0,0,0,1,0,0,1,0,0,0,0,1,1,0,1,0,0,0,0,0,1,0,0,0,0,0,0,0,0,0,0,1,1,0,0,0,0,1,0,1,0,0,0,1,0,0,1,0,0,0,0,0,0,0,0,0,0,0,0,0,0,1,1,0,0,0,0,0,0,1,0,0,1,1,0,0,0,0,0,1,0,0,0,0,0,0,0,0,0,0,0,0,0,0,0,0,0,0,1,0,0,1,0,1,0,1,0,0,1,0,0,0,0,0,0,0,0,0,0,0,0,0,0,1,0,0,0,0,0,0,0,0,1,0,1,0,0,0,0,1,1,0,0,0,0,0,0,0,0,0,0,1,0,0,1,0,0,1,0,0,0,0,0,1,0,0,0,0,0,0,0,0,1,0,0,0,0,0,0,0,1,0,0,0,0,0,0,0,0,0,0,0,0,1,0,1,1,0,0,0,0,0,0,0,0,0,0,1,0,0,0,1,0,0,0,0,0,0,0,1,0,1,0,0,0,0,0,0,0,0,0,0,0,1,0,0,1,0,0,0,0,0,0,1,0,0,0,0,0,0,0,0,0,0,1,0,0,0,0,0,1,0,0,1,0,0,0,0,0,0,0,0,0,1,1,0,0,1,0,0,0,0,0,0,0,0,0,1,0,0,0,0,0,0,0,0,0,0,0,0,0,1,0,0,0,0,1,0,0,0,1,0,1,0,0,0,0,0,0,0,1,0,0,1,0,0,1,0,0,0,0,1,0,0,0,1,0,0,0,0,1,1,0,0,0,0,1,0,0,0,0,0,0,0,1,1,1,0,0,0,1,0,0,0,1,0,0,0,0,1,0,0,0,0,0,0,0,0,0,0,0,0,0,0,0,0,0,0,0,0,0,0,0,0,0,0,0,0,1,0,0,0,1,0,0,0,0,0,1,1,0,0,1,1,0,0,0,0,0,0,0,0,0,0,0,0,0,0,0,0,0,0,0,0,0,1,0,0,1,0,1,0,0,0,1,0,0,1,0,0,0,0,0,0,0,0,1,0,0,0,1,0,0,1,0,1,0,0,0,0,0,0,0,0,0,1,0,0,0,0,0,0,0,0,0,0,0,1,0,0,1,0,0,0,0,0,1,0,0,0,1,1,0,0,0,0,0,0,0,0,1,0,0,0,0,1,0,0,0,0,0,1,0,0,0,1,0,0,1,0,0,0,1,0,0,0,0,0,0,1,0,0,0,0,0,0,0,1,0,0,0,1,0,0,1,0,0,0,0,1,0,0,0,1,0,1,1,0,0,0,0,0,0,0,0,0,0,0,1,0,0,1,0,0,0,0,0,0)
